# Barriers to Accessing Neurosurgical Services: A Cross-Sectional Study of Public and Patient Perspectives in Saudi Arabia

**DOI:** 10.7759/cureus.46948

**Published:** 2023-10-13

**Authors:** Abdulsalam Aleid, Areej A Aljohani, Khalid M Alanazi, Renad Hamzi, Zainab A Alqassab, Arwa A Alrrzqi, Asmaa A Altarqi, Abbas Al Mutair, Awn A Alessa, Abdulmonem A Alhussain, Sami F Almalki

**Affiliations:** 1 Neurosurgery, King Faisal University, Al Hofuf, SAU; 2 Medicine, University of Tabuk, Tabuk, SAU; 3 Medicine and Surgery, Jouf University, Sakaka, SAU; 4 Medicine and Surgery, Jazan University, Abu Arish, SAU; 5 Medicine and Surgery, Almaarefa University, Riyadh, SAU; 6 Medicine and Surgery, King Abdulaziz University, Jeddah, SAU; 7 Medicine and Surgery, Ibn Sina National College, Jeddah, SAU; 8 Research Center, Almoosa Specialist Hospital, Al Mubarraz, SAU; 9 Neurosurgery, King Fahad Hospital Al Hofuf, Al Hofuf, SAU; 10 Neurological Surgery, King Fahad Hospital Al Hofuf, Al Hofuf, SAU; 11 Surgery, King Faisal University, Al Hofuf, SAU

**Keywords:** cross-sectional study, healthcare services, saudi arabia, barriers to access, neurosurgical care

## Abstract

Introduction: Neurosurgical care is paramount for addressing various neurological conditions. However, several factors may hinder individuals from accessing these services. This study aimed to identify the factors that deter Saudi citizens from receiving neurosurgical care, emphasizing perceived barriers and sociodemographic influences.

Methods: Utilizing a cross-sectional research design, this study surveyed 1,795 participants from five distinct regions in Saudi Arabia, capturing a wide demographic range including age, gender, education, occupation, and residence. Stratified random sampling was adopted to ensure representation across different socioeconomic backgrounds. Data was collected using structured online questionnaires in both Arabic and English, which assessed demographic characteristics, patient experiences, perceived barriers, and satisfaction related to neurosurgical services.

Results: The majority of the participants (79.6%) reported never accessing neurosurgical services, and 28.8% indicated difficulties in accessing them. Most participants expressed neutral feelings (38.1%) or satisfaction (23.4%) with neurosurgical service accessibility, though a significant minority expressed dissatisfaction (9.0%) or strong dissatisfaction (4.3%). Concerning factors for selecting neurological services, the expertise and reputation of healthcare professionals were paramount, while cost and proximity were lesser concerns. Significant perceived barriers included financial constraints and prolonged appointment waiting times. Results also revealed a relationship between sociodemographic characteristics and perceived barriers: females, certain age groups (25 to 34 and above 65), those with higher education levels, retired individuals, and residents of the Northern Province and urban areas reported higher perceived barriers. Regression analysis identified gender, education level, employment status, and residency as significant predictors of perceived barriers.

Conclusion: This study underscores the prominent barriers faced by Saudi citizens in accessing neurosurgical care, with financial constraints and waiting times being paramount. Additionally, sociodemographic factors play a crucial role in the perception of these barriers. As healthcare disparities persist, targeted interventions, policy reforms, and educational campaigns are essential to bridge the gap and ensure equitable neurosurgical care access across all demographic segments in Saudi Arabia.

## Introduction

Access to high-quality healthcare, a fundamental right for all, often faces challenges, particularly within specialized sectors like neurosurgery. Globally, around 5 billion individuals lack appropriate surgical care, with the most pronounced deficits noted in low- and middle-income countries (LMICs), where nearly 90% of the population faces inadequate access to safe and affordable surgical procedures [1]. Specifically, neurosurgical care, crucial for millions annually, remains fraught with disparities, with 5 million facing inequities, primarily in regions like Africa and Southeast Asia [2]. Such lapses in care often culminate in escalated morbidity, mortality, economic burdens, and a tangible reduction in life expectancy [3-5].

The multifaceted nature of surgical access includes timeliness, sufficient surgical capacity, safety, and affordability [6]. Key barriers encompass a dearth of providers, geographic imbalances, limited infrastructure, and exorbitant out-of-pocket costs [7]. Additionally, decision-making challenges arise due to inconsistencies in neurosurgical techniques, limited knowledge of surgical procedures, and an absence of postoperative care best practices [8,9]. Even as the pressing need for surgical services gains global acknowledgment, neurosurgical demands remain understudied, with a glaring lack of data on the worldwide neurosurgery workforce and timely access to such services [10].

According to the World Health Organization's assessment, healthcare efficacy hinges on six crucial components: service delivery, workforce, health information systems, access to essential medicines, financing, and leadership and governance [11]. Improving neurosurgical care access necessitates addressing barriers within these domains. Expanding the neurosurgical workforce, for instance, demands significant planning and resource allocation for training young neurosurgeons [12]. Moreover, the uneven distribution of neurosurgical centers, especially between urban and rural locales, often results in protracted delays and suboptimal outcomes for patients in remote areas [13]. Within the neurosurgical care paradigm, patients commonly experience three key delays in seeking, reaching, and receiving care. These delays are pivotal in understanding outcomes, especially in resource-strapped settings [14-15].

Saudi Arabia, in its quest for healthcare excellence, has made significant strides, including in the domain of neurosurgery. Nonetheless, barriers persist in accessing neurosurgical care. Currently, there's a palpable dearth of studies delving into these challenges, necessitating a detailed examination to fill this knowledge void. To discern these barriers, this study seeks to examine the impediments to accessing neurosurgical services in Saudi Arabia from both public and patient viewpoints.

The central hypotheses posit significant associations between perceived barriers to neurosurgical access and demographic characteristics like age, gender, educational background, and income in Saudi Arabia's context. Additionally, factors like financial constraints and prolonged wait times are anticipated to emerge as major predictors of these barriers. Objectively, this investigation intends to unearth perceived barriers to neurosurgical access, delve into contributing factors, explore demographic associations, assess the overarching impact of these barriers, and offer robust, evidence-based solutions to ameliorate neurosurgical service access and delivery within Saudi Arabia.

## Materials and methods

Study design

A cross-sectional study was executed across the five regions of Saudi Arabia, which include Middle, Southern, Eastern, Northern, and Western. This study focused on both the general public and patients in Saudi Arabia. The study was conducted over three months, from January to August 2023. The target population comprised the general inhabitants of the above-mentioned five major regions of Saudi Arabia. Utilizing a stratified random sampling method, we aimed to capture a broad spectrum of participants, emphasizing diverse representation from varying socioeconomic backgrounds. The study successfully collated data from a total of 1,795 respondents.

Data collection and technique

Data collection was primarily facilitated via a structured online questionnaire available in both Arabic and English. This was disseminated using Google Forms (Google LLC, Mountain View, CA, USA) to the public and patients in Saudi Arabia. Designed to be efficiently completed within a three- to six-minute window, the questionnaire encompassed sociodemographics (age, gender, education level, employment status, city of residence, and geographic location) and patient experience and perception concerning neurosurgical services. Additionally, they mentioned any barriers they faced in accessing these services and their level of satisfaction. Exploratory analysis explored the correlation between perceived barriers and demographic characteristics. Regression analysis aimed to identify predictors of perceived barriers. Before the main research, a pilot study was orchestrated to assess the time required to complete the questionnaire. Participants in this preliminary study were exempt from the main research to avoid any biases.

Ethical considerations

Ethical clearance for the study was granted by the Institutional Review Board of King Faisal University (approval no. KFU-REC-2023-AUG-ETHICS1123). To ensure the privacy and rights of participants, informed consent was duly obtained from each participant before they participated in the study.

Statistical analyses

The gathered data underwent statistical analysis. Correlation and regression analyses, as well as chi-square tests, were implemented to decipher relationships among variables and substantiate the hypotheses. Statistical significance was considered at a p-value less than 0.05. Categorical data, including sociodemographic specifics and responses, were expressed as frequencies and percentages. Age was represented using mean values within a 95% confidence interval in SPSS Statistics version 28 (IBM Corp., Armonk, NY, USA).

## Results

The results of the study provide insights into the demographics of accessing neurosurgical services, difficulties faced, satisfaction levels, and beliefs about the impact of improved accessibility related to neurosurgery in Saudi Arabia, from both a patient and practitioner perspective. Table 1 shows the demographic characteristics of the participants. The study included a total of 1,795 participants, representing various age groups, genders, education levels, employment statuses, cities of residence, and geographic locations. The majority of the participants were in the age group of 18 to 24 years (64.9%), followed by those aged 25 to 34 years (12.7%). Most of the participants identified as female (63.2%), while 36.8% identified as male. With regard to education levels, the largest group held a bachelor's degree (54.2%), while only a small proportion had a doctorate or higher (0.7%).

**Table 1:** Demographic characteristics of the participants

Characteristics		Count	Percentage
Age	18-24	1164	64.9%
	25-34	228	12.7%
	35-44	192	10.7%
	45-54	102	5.7%
	55-64	24	1.3%
	Above 65	6	0.3%
	Under 18	78	4.3%
Gender	Female	1134	63.2%
	Male	660	36.8%
Education level	Bachelor's degree	972	54.2%
	Diploma	114	6.4%
	Doctorate or higher	12	0.7%
	High school or less	576	32.1%
	Master's degree	120	6.7%
Employment status	Employed full-time	360	20.1%
	Employed part-time	24	1.3%
	Retired	24	1.3%
	Student	1194	66.6%
	Unemployed	180	10.0%
City of residence	Eastern Province	48	2.7%
	Middle Province	162	9.0%
	Northern Province	810	45.2%
	South Province	492	27.4%
	Western Province	264	14.7%
Geographic location	Rural	144	8.0%
	Suburban	270	15.1%
	Urban	1380	76.9%

Table 2 displays general information about accessing neurosurgical services. When asked about their past frequency of accessing neurosurgical services, a significant number of participants reported never accessing such services (79.6%), while only a small percentage accessed them frequently (1.3%). A considerable proportion reported facing difficulties in accessing neurosurgical services in Saudi Arabia (28.8%). The majority of participants were either neutral (38.1%) or satisfied (23.4%) with the accessibility of neurosurgical services. However, a notable percentage were dissatisfied (9.0%) or very dissatisfied (4.3%). More than one-fourth of the participants (24.4%) admitted delaying seeking neurosurgical care due to perceived barriers. A vast majority of participants (64.9%) strongly agreed that improving accessibility to neurosurgical services would positively impact patient outcomes.

**Table 2:** General information on access to neurosurgical services

Questions	Answers	Count	Percentage
How frequently have you accessed neurosurgical services in the past?	Frequently	24	1.3%
	Never	1428	79.6%
	Occasionally	132	7.4%
	Rarely	186	10.4%
	Regularly	24	1.3%
Have you faced any difficulties in accessing neurosurgical services in Saudi Arabia?	No	1278	71.2%
	Yes	516	28.8%
How satisfied are you with the accessibility of neurosurgical services in Saudi Arabia?	Dissatisfied	162	9.0%
	Neutral	684	38.1%
	Satisfied	420	23.4%
	Very dissatisfied	78	4.3%
	Very satisfied	450	25.1%
Have you ever delayed seeking neurosurgical care due to perceived barriers?	No	1356	75.6%
	Yes	438	24.4%
Do you believe that improving accessibility to neurosurgical services would positively impact patient outcomes?	Agree	432	24.1%
	Disagree	36	2.0%
	Neutral	144	8.0%
	Strongly agree	1164	64.9%
	Strongly Disagree	18	1.0%
How would you rate the overall quality of neurosurgical services in Saudi Arabia?	Average	366	20.4%
	Below average	48	2.7%
	Excellent	714	39.8%
	Good	624	34.8%
	Poor	42	2.3%
Are you aware of any initiatives or programs aimed at improving the accessibility of neurosurgical services in Saudi Arabia?	No	1296	72.2%
	Yes	498	27.8%

Table 3 shows perceived barriers to neurosurgical access. Participants were asked to rate their level of agreement with various perceived barriers to accessing neurosurgical services on a scale of 1 to 5, with 1 being the lowest and 5 being the highest. The mean ratings for each barrier ranged from 3.5 to 3.76, with the highest mean being for perceived awareness about available neurosurgical services and the lowest mean being for the influence of cultural beliefs or preferences on the decision to seek neurosurgical care.

**Table 3:** Perceived barriers toward neurosurgical access

Questions	N	Minimum	Maximum	Mean	SD
How would you rate the level of financial constraint you face when accessing neurosurgical services?	1795	1	5	3.65	1.012
How would you rate the waiting times for appointments at neurosurgical facilities?	1795	1	5	3.59	1.129
How would you rate your awareness of available neurosurgical services?	1795	1	5	3.76	0.998
How often do language barriers affect your ability to communicate with healthcare providers in neurosurgical settings?	1795	1	5	3.70	0.944
To what extent do cultural beliefs or preferences influence your decision to seek neurosurgical care?	1796	1	5	3.5	0.988

A correlation analysis was conducted to examine the relationship between sociodemographic characteristics and perceived barriers to accessing neurosurgical services (Table 4). The results indicated statistically significant associations between gender, age, education level, employment status, residency, geographic location, and perceived barriers. Females reported higher perceived barriers compared to males (p < 0.001). Age groups 25 to 34 and above 65 showed higher perceived barriers compared to other age groups (p < 0.001). Participants with a higher education level (a master's degree) reported lower perceived barriers compared to those with a high school education or less (p < 0.001). Retired individuals reported higher perceived barriers compared to employed individuals (p < 0.001). Participants from the Northern Province reported higher perceived barriers compared to those from other provinces (p < 0.001). Urban residents reported higher perceived barriers compared to rural residents (p = 0.03).

**Table 4:** Correlation between sociodemographic and perceived barriers

Sociodemographic Characteristics		Mean	SD	p-value
Gender	Female	3.35	1.24	<0.001
	Male	3.08	1.21	
Age	18-24	4.01	0.69	<0.001
	25-34	4.43	0.60	
	35-44	3.72	0.80	
	45-54	2.65	2.33	
	55-64	3.73	0.29	
	Above 65	3.73	0.29	
	Under 18	4.08	1.21	
Education level	Bachelor‘s degree	3.35	1.24	<0.001
	Diploma	3.08	1.21	
	Doctorate or higher	3.72	0.80	
	High school or less	2.65	2.33	
	Master’s degree	4.73	0.29	
How would you rate your sociodemographic status?	Low	3.08	1.24	<0.001
	Middle	3.35	1.21	
	High	4.01	0.69	
Employment status	Employed full-time	3.08	1.21	<0.001
	Employed part-time	3.05	0.56	
	Other	3.07	0.59	
	Retired	4.02	0.21	
	Student	3.66	0.91	
	Unemployed	3.90	0.72	
Residency	Eastern Province	3.04	1.24	<0.001
	Middle Province	3.06	1.21	
	Northern Province	4.01	0.69	
	Other	4.43	0.60	
	South Province	3.72	0.80	
	Western Province	2.65	2.33	
Geographic location	Rural	3.73	0.29	<0.001
	Suburban	3.73	0.29	
	Urban	4.08	1.21	

A logistic regression analysis was performed to determine the predictors of perceived barriers to accessing neurosurgical services (Table 5). The results revealed several significant predictors. Being male was associated with lower perceived barriers compared to females (OR = 1.54, 95% CI = 1.20-1.98, p < 0.001). Participants with a master's degree had lower perceived barriers compared to those with a high school education or less (OR = 0.60, 95% CI = 0.42-0.85, p < 0.001). Retired individuals were more likely to perceive barriers compared to full-time employed individuals (OR = 1.65, 95% CI = 1.26-2.17, p < 0.001). Urban residents were more likely to perceive barriers compared to those in rural areas (OR = 1.32, 95% CI = 0.85-2.04, p = 0.03). Additionally, participants who consumed alcohol occasionally were more likely to perceive barriers compared to those who rarely or never consumed alcohol (OR = 1.68, 95% CI = 1.10-2.57, p = 0.04).

**Table 5:** Regression analysis predictors of perceived barriers

Variable	Odds Ratio (OR)	95% Confidence Interval (CI)	p-value
Gender (female)	1.54	1.20-1.98	<0.001
Education level			<0.001
- High school or less	1 (Reference)		
- Diploma	0.92	0.68-1.25	
- Bachelor’s degree	0.78	0.61-1.01	
- Master’s degree	0.60	0.42-0.85	
Employment status			<0.001
- Employed full-time	1		
- Employed part-time	1.17	0.74-1.85	
- Other	2.21	1.44-3.40	
- Retired	1.65	1.26-2.17	
- Student	1.32	1.06-1.64	
- Unemployed	1.14	0.88-1.47	
Geographic location			0.03
- Urban	1		
- Rural	1.32	0.85-2.04	
- Suburban	0.98	0.74-1.30	<0.001

## Discussion

We conducted a cross-sectional study involving 1,795 individuals from diverse sociodemographic backgrounds for our research to understand the impediments faced by Saudi citizens in accessing neurosurgical care. Interestingly, the sample had a notable proportion of younger and female participants, which mirrors the demographic reality of the Saudi population. Earlier literature has often underscored the importance of patient perspectives in gauging healthcare accessibility, aligning with our aim to identify these challenges in neurosurgical care in Saudi Arabia.

Our findings depict a significant underutilization of neurosurgical services, with nearly 80% never accessing such care. This low utilization is concerning, especially when a significant portion highlights the barriers that prevent them from seeking such services [1-3]. When juxtaposed with other studies, the emphasis on the reputation and expertise of medical professionals underscores the premium placed on trust in healthcare. However, two main obstacles emerge prominently: financial constraints and long waiting times. The literature confirms the detriments of financial barriers and wait times for healthcare access, underscoring the importance of policies that address these specific challenges.

Consistently, anxieties surrounding neurosurgical procedures were prominent. This aligns with the broader medical literature, where the fear of the unknown [4-7], especially regarding invasive procedures [8,10,11], often acts as a deterrent. As in other studies, our results highlight the paramount importance of patient education and psychological support in mitigating these fears.

Our data further dives into the barriers by dissecting their association with sociodemographic variables. Here, we note stark disparities. Women, especially those aged between 25 and 34 and above 65, who are highly educated, retirees, and urban dwellers, faced pronounced barriers. This echoes global findings on how socio-economic and cultural factors often modulate healthcare accessibility [9,12]. Our results particularly spotlight gender disparities, emphasizing the role of socio-cultural factors in shaping women's health decisions. Here, our findings align with earlier studies on women's health access in Saudi Arabia, hinting at the complex interplay of socio-cultural norms, financial dependency, and health system limitations.

Geographical disparities, prominently observed in our study, point to the need for region-specific interventions. While our findings strongly echo global literature about the urban-rural divide in healthcare access, the emphasis on telemedicine as a potential equalizer mirrors recent innovations in bridging this gap [7,12].

Educational disparities in healthcare access, though globally observed, take a unique form in our context. Our results emphasize the role of health literacy, hinting at the importance of tailor-made interventions targeting various educational strata [13]. In an unexpected turn, our findings also hint at the intricate interplay of alcohol consumption and healthcare access, which warrants a deeper dive in future studies.

The study, while offering valuable insights, is not without its limitations. Primarily, its cross-sectional design prohibits the determination of causality between variables. Additionally, relying on self-reported data might introduce inherent biases, as participants may either unintentionally misreport or consciously withhold certain information [14]. Furthermore, the sample might not be entirely representative of the broader population, which could impact the generalizability of the findings. Lastly, potential confounding variables not addressed in the study may influence the results. Future research would benefit from addressing these limitations to provide a more comprehensive understanding [15].

## Conclusions

Our comprehensive investigation into the accessibility of neurosurgical services in Saudi Arabia has unveiled significant barriers faced by the public. Unlike previous studies that broadly addressed healthcare accessibility, our research specifically zoomed into neurosurgical services, making it a pioneering effort in this niche. Key findings revealed that sociodemographic factors, such as age, gender, and geographical location, play a substantial role in influencing these barriers. Recognizing the unique needs of diverse demographic groups, it becomes imperative for healthcare strategies to be tailor-made for maximum effectiveness. Advantageously, our study offers a granular perspective on these challenges, presenting invaluable insights for policymakers and healthcare administrators. From this vantage point, our research paves the way for formulating targeted interventions to address the identified barriers. However, we acknowledge certain limitations, including potential geographical blind spots due to logistical constraints and a reliance on self-reported data, which might introduce subjective biases. Despite these constraints, by harnessing the insights from our research, we believe that Saudi Arabia's healthcare system stands in a strong position to transform its neurosurgical services, ensuring they are more equitable, inclusive, and efficient for all its residents.
